# Small-Angle Neutron Scattering by the Magnetic Microstructure of Nanocrystalline Ferromagnets Near Saturation

**DOI:** 10.6028/jres.104.019

**Published:** 1999-06-01

**Authors:** J. Weissmüller, R. D. McMichael, A. Michels, R. D. Shull

**Affiliations:** Institut für Nanotechnology, Forschungszentrum Karlsruhe, 76021 Karlsruhe, Germany; Universität des Saarlandes, 66041 Saarbrücken, Germany; National Institute of Standards and Technology, Gaithersburg, MD 20899-0001, USA; Universität des Saarlandes, 66041 Saarbrücken, Germany; National Institute of Standards and Technology, Gaithersburg, MD 20899-0001, USA

**Keywords:** exchange constant, magnetic anisotropy, magnetic microstructure, magnetism, micromagnetics, nanocrystalline materials, neutron scattering, small-angle neutron scattering, spin-wave stiffness

## Abstract

The paper presents a theoretical analysis of elastic magnetic small-angle neutron scattering (SANS) due to the nonuniform magnetic microstructure in nanocrystalline ferromagnets. The reaction of the magnetization to the magnetocrystalline and magnetoelastic anisotropy fields is derived using the theory of micromagnetics. In the limit where the scattering volume is a single magnetic domain, and the magnetization is nearly aligned with the direction of the magnetic field, closed form solutions are given for the differential scattering cross-section as a function of the scattering vector and of the magnetic field. These expressions involve an anisotropy field scattering function, that depends only on the Fourier components of the anisotropy field microstructure, not on the applied field, and a micromagnetic response function for SANS, that can be computed from tabulated values of the materials parameters saturation magnetization and exchange stiffness constant or spin wave stiffness constant. Based on these results, it is suggested that the anisotropy field scattering function *S_H_* can be extracted from experimental SANS data. A sum rule for *S_H_* suggests measurement of the volumetric mean square anisotropy field. When magnetocrystalline anisotropy is dominant, then a mean grain size or the grain size distribution may be determined by analysis of *S_H_*.

## 1. Introduction

Nanocrystalline ferromagnets exhibit favorable soft [1,2] and hard magnetic [3,4,5] properties that are the subject of current research. In the simplest conceptual case, these materials can be single phase, single component polycrystalline solids with a grain size of the order of 10 nm, and with uniform values of the local magnetization magnitude and of the exchange stiffness constant. In this case, the nuclear microstructure affects the magnetization and, hence, magnetic properties such as coercivity and remanence, exclusively through the magnetic anisotropy. The nuclear microstructure determines the magnitude and the local orientation of the *anisotropy field*, that is the derivative of the magnetic anisotropy energy density with respect to the orientation of the magnetization, which acts as a torque on the magnetic moments, resulting in a nonuniform magnetic microstructure. Because of the importance of the anisotropy fields for the magnetic properties of nanocrystalline materials, it is of interest to characterize their magnitude and spatial arrangement, in other words the microstructure of the anisotropy fields. In this paper, we show how quantitative information on the anisotropy field microstructure can be obtained by combining small-angle neutron scattering (SANS) data with an analysis in terms of the theory of micromagnetics.

The magnetic domain structure of materials at low applied magnetic fields gives rise to neutron *refraction* [6] and to *depolarization* [7,8] of the transmitted neutron beam, and can be studied thereby, but of interest here is neutron *scattering* at sufficiently high applied fields where the scattering volume is essentially a single magnetic domain. In this case, magnetic SANS arises from small (static) variations, on the scale of a few nanometers to a few hundreds of nanometers, of the orientation of the magnetization vector about the direction of the applied field. The technique is therefore well suited for combination with micromagnetics theory [9,10,11], that describes the spatial variation of the magnetization, at equilibrium, in terms of a continuum approach which applies to length scales where the discrete atomic structure of matter can be neglected. In amorphous ferromagnets, *inelastic* SANS is an established technique for determining an important parameter of micromagnetics, the spin-wave stiffness constant [12,13]. *Elastic* SANS, that is of interest in the present context, has been shown to yield information on magnetic correlations in superparamagnetic nanocomposites [14], and on the ferromagnet-superparamagnet transition which occurs near the Curie temperature of one of the phases in multi-phase nanocrystalline ferromagnets [15]. In single-phase nanocrystalline ferromagnets, SANS indicates the presence of correlations in the magnetic structure on a length-scale larger than the grain size [16, 17]. Studies of dislocation arrays in cold-worked ferromagnetic single crystals have demonstrated that a combination of SANS experiments with micro-magnetics theory can provide information on the nuclear microstructure [18]. Preliminary results of the present study [19] indicated that SANS experiments on single-phase bulk nanocrystalline materials with low porosity, hence low nuclear scattering cross-section, are in good agreement with predictions from micromagnetics over a wide range of applied magnetic fields and scattering vectors.

Modeling remanence or coercivity of nanocrystalline materials by micromagnetics requires numerical computation [5]. But in the limit of nearly parallel alignment of all spins, at sufficiently high applied fields, there are closed form solutions for the magnetization [10,21] that are amenable to combination with scattering theory, and it is this approach that we shall explore. The paper is organized as follows: Sec. 2 discusses the micromagnetics solution for the Fourier components of the magnetization in terms of the applied magnetic field and of the Fourier components of the anisotropy field. Section 3 combines the results of micromagnetics with the theory of magnetic neutron scattering, and derives a result for the differential scattering cross-section in terms of the Fourier components of the anisotropy field and of a micromagnetics response function for SANS that depends on the applied field and on measurable magnetic materials constants. Section 4 is a derivation of expressions for averages of the scattering cross-section that apply to commonly used experimental scattering geometries and to materials with isotropic microstructure. Section 5 discusses an invariant of magnetic SANS, that relates to measurement of the magnitude of the anisotropy field. Section 6 deals specifically with the special case of nanocrystalline materials where the anisotropy field is exclusively from magnetocrystalline anisotropy. Section 6.1 discusses an approximate closed form solution for the magnetization in real space, both for a single grain and for a nanocrystalline material. Based on this solution, criteria are derived for the range of grain size and applied field to which the result of the previous sections apply. Section 6.2 presents results of the theory for SANS of nanocrystalline materials. Experimental results on nanocrystalline Ni and Co will be presented in a subsequent publication [20].

## 2. Micromagnetics

We aim to analyze the magnetic microstructure in a bulk nanocrystalline material, that is a space-filling array of nanometer-sized grains with different crystallographic orientations, restricting attention to situations where elements of the nuclear microstructure, such as grain boundaries or dislocations, do not affect the local values of the atomic magnetic moment and of the exchange stiffness constant. With this in mind, we consider the saturation magnetization *M*_S_ = *ρ*_a_ *μ*_a_ and the exchange stiffness constant *A* to be uniform. The symbols *ρ*_a_ and *μ*_a_ denote the atomic density and the atomic magnetic moment, respectively. The inhomogeneous nuclear microstructure affects the magnetization because the combined effects of magnetocrystalline and magnetoelastic anisotropy determine the anisotropy energy density *a* that depends on position ***x*** and on the magnetization ***M***, that is *a* = *a*[***x***, ***M***(***x***)]. The anisotropy energy enters the equations of micromagnetics theory through the *anisotropy field* (or perturbing field) ***H***_P_(***x***), defined (in SI units, and with *μ*_0_ the magnetic constant, also called the permeability of vacuum) by

$$H_{P}=-\mu_{0}^{-1}\partial a/\partial M.$$ (1)

With ***M*** subject to |***M***| = *M*_S_, the vector ***H***_P_ is normal to ***M***; in other words, the anisotropy field results in a torque on the magnetic moments, of nonuniform magnitude and direction, that deflects the magnetic moments from the perfectly aligned state.

At equilibrium, the static response of the magnetization to the magnetic field ***H*** and to the anisotropy field satisfies the micromagnetics equation (compare to Sec. 4.1 of Ref. 9):

$$[l_{M}^{2}\{\nabla^{2}M_{x},\nabla^{2}M_{y},\nabla^{2}M_{z}\}+H+H_{P}]\times M=0$$ (2)

for an orthonormal basis {***e****_x_*, ***e****_y_*, ***e****_z_*}, where for any vector ***f*** the scalars *f_x_, f_y_, f_z_*, and *f* are, respectively, the Cartesian coordinates of ***f*** relative to {***e****_x_*, ***e****_y_*, ***e****_z_*} and the modulus of ***f***. The *magnetostatic exchange length* [5,23] *l_M_* is defined as

$$l_{M}=\sqrt{\frac{2A}{\mu_{0}\;M_{S}^{2}}}.$$ (3)

In the limit where the angle of misalignment of the magnetic moments relative to the mean magnetization 〈***M***〉 is small, Eq. (2) can be linearized [9] by neglecting terms that are of second order in ***M***_P_(***x***), the component of the magnetization perpendicular to 〈***M***〉,

$$M_{P}(x)=M(x)-\langle M\rangle .$$ (4)

It has been shown [10,21] that, with the magnetization and the fields expressed in terms of their Fourier transforms, the linearized equation can be solved independently for each wavevector ***q***. We find it useful to discuss the solution in terms of ***h***(***q***), the Fourier transform of ***H***_P_(***x***), and of ***m***(***q***), the Fourier transform of ***M***_P_(***x***)/*M*_S_:

$$H_{P}(x)={\left(2\pi \right)}^{-3/2}\int \int \int_{-\infty }^{\infty }h(q)\;\exp\;(-i\mathrm{qx})\;d^{3}q,$$ (5)

$$M_{P}(x)/M_{S}={\left(2\pi \right)}^{-3/2}\int \int \int_{-\infty }^{\infty }m(q)\;\exp\;(-i\mathrm{qx})\;d^{3}q.$$ (6)

By definition, ***H***_P_ depends not only on ***x*** but also on ***M*** and, hence, on the applied magnetic field. For instance, ***H***_P_ vanishes when the magnetization is aligned with one of the low energy (“easy”) directions of the crystal lattice. In the limit of small misalignment, changes of ***H***_P_ due to re-orientation of ***M*** result only in second order effects on the magnetization; therefore, the dependency of ***H***_P_ on ***M*** can be ignored.

We write the magnetic field ***H*** as the sum of the applied field ***H***_a_ and of the demagnetizing field ***H***_d_, and separate ***H***_d_ into two components: the field, ***H***_d_^s^, which arises from the discontinuity of ***M*** at the macroscopic sample surface, and the field, $H_{d}^{b}$, which arises from the divergence of ***M*** in the bulk. $H_{d}^{s}$ varies slowly with position in the material and is here approximated by the uniform field $H_{d}^{s}=-N_{d}\langle M\rangle$, with the demagnetizing factor *N*_d_ dependent on the sample geometry. It is emphasized that, as a consequence of the uniformity of *M*_S_ and *A*, there is no *a priori* discontinuity of ***M*** at internal interfaces, therefore $H_{d}^{s}$ is exclusively from the macroscopic external surface of the material, and is entirely unrelated to the grain size or grain shape. Grain shape enters the equations of micromagnetics through ***H***_P_(***x***), and its effect on magnetic properties is therefore accounted for in the solution for ***M***(***x***).

The bulk contribution to ***H***_d_ is given by (compare Ref. 21)

$$\begin{array}{c} H_{d}^{b}(x)=-M_{S}(2\pi {)}^{-3/2}\int \int \int_{-\infty }^{\infty }\frac{[m(q)\cdot q]q}{q^{2}}\\ \;\exp(-i\mathrm{qx})\;d^{3}q; \end{array}$$ (7)

it gives rise to a restoring force that tends to suppress variations of ***M*** with Fourier components ***m***(***q***) parallel ***q***, thus stiffening the magnetic microstructure against such variations.

Solutions to Eq. (2) have been derived for several special cases, in particular for amorphous ferromagnets with random anisotropy (ignoring $H_{d}^{b}$) [22], and for the case where ***H***_P_(***x***) is due to magnetostriction in cubic single crystals, for instance in the strain field of a dislocation [10]. For the present case of a polycrystal, where a more general form of the magnetocrystalline anisotropy field is considered, it is readily verified by insertion that, in the limit of small misalignment, the solution is

$$m(q)=\frac{h(q)}{H_{\mathrm{eff}}+M_{S}\;\sin^{2}\theta }+\frac{M_{S}}{H_{\mathrm{eff}}}\frac{q_{⊥}\times [h(q)\times q_{⊥}]}{q^{2}(H_{\mathrm{eff}}+M_{S}\;\sin^{2}\theta )}.$$ (8)

The vector ***q***_'_ denotes the component of ***q*** that is normal to the applied field ***H***_a_, and *θ* is the angle between ***q*** and ***H***_a_. *H*_eff_ denotes an *effective field*, defined by

$$H_{\mathrm{eff}}=H_{i}(1+{l_{H}}^{2}q^{2}).$$ (9)

*H*_eff_ depends on the magnitude of the *internal field* $H_{i}=H_{a}+H_{d}^{s}$ and on the *exchange length of the internal field* [23], *l_H_*, defined by

$$l_{H}=\sqrt{\frac{2A}{\mu_{0}\;M_{S}\;H_{i}}}.$$ (10)

A simpler expression than Eq. (8) is relevant for the most commonly used geometry for SANS (compare Sec. 3 below). Consider the applied field and the mean magnetization along ***e****_z_*, hence the anisotropy field in the plane containing ***e****_x_* and ***e****_y_.* When the incident neutron wavevector ***k***_0_ is along ***e****_x_*, perpendicular to ***H***_a_, then the scattering vector for elastic SANS is in the plane containing ***e****_y_* and ***e****_z_*. Therefore, only Fourier components of the magnetization with *q_x_* = 0 are probed with this SANS geometry. For these components, Eq. (8) simplifies to

$$m_{x}(q)=\frac{h_{x}(q)}{H_{\mathrm{eff}}},\;m_{y}(q)=\frac{h_{y}(q)}{H_{\mathrm{eff}}+M_{S}\;\sin^{2}\theta },\;m_{z}(q)=0.$$ (11)

The term that depends on *u* in Eq. (11) originates from the demagnetizing field from divergence of ***M*** in bulk, $H_{d}^{b}$. At high applied fields this term is small compared to ***H***_eff_, so that the Fourier coefficient ***m***(***q***) of the magnetization is essentially the product of the Fourier coefficient ***h***(***q***) of the anisotropy field and of the reciprocal of the effective field. Because of the convolution theorem, the product in reciprocal space corresponds in real space to a convolution with the Fourier transform of 1/*H*_eff_, which is a decaying exponential with a characteristic length *l_H_*. The central implication of the result, Eqs. (8)–(10), is therefore that, within the assumptions of uniformity and linearity, the magnetic microstructure is the convolution of the anisotropy field microstructure with an exponential response function with a characteristic length *l_H_* that varies as the reciprocal root of the internal field.

**Fig. 1:**
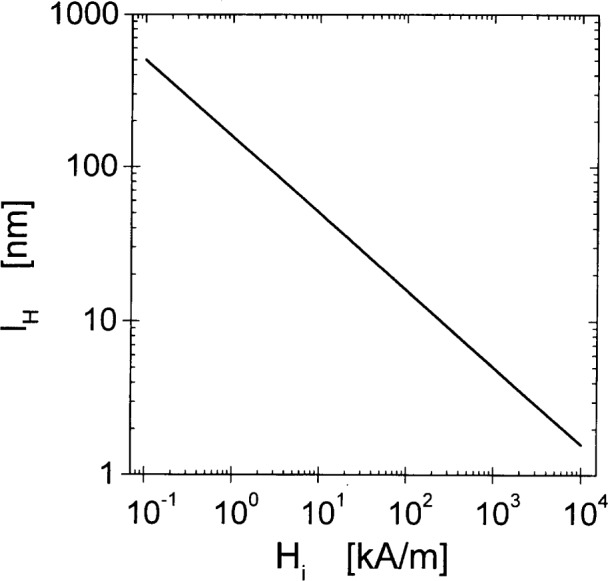
Exchange length *l_H_* versus internal magnetic field *H*_i_ for Ni.

**Fig. 2:**
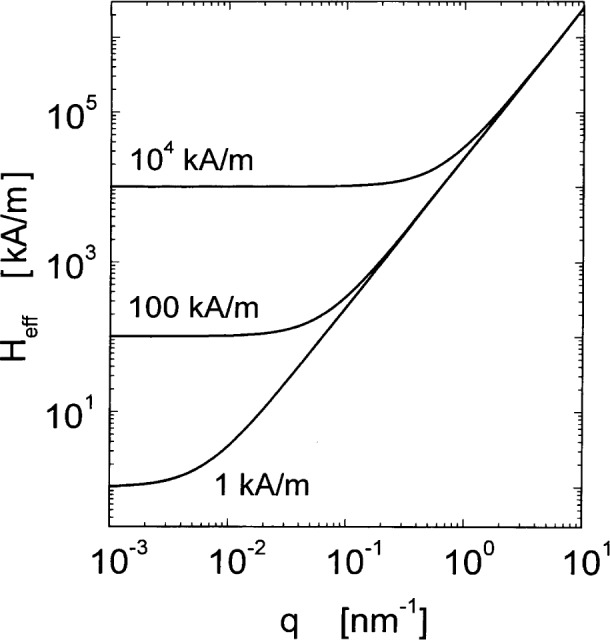
Effective field *H*_eff_ for Ni versus wavevector ***q***. The numbers in the figure indicate the value of the magnetic field *H*_i_.

For the purpose of illustration, we shall repeatedly refer to the example of Ni. We denote by *μ*_B_, *D*, and *g* the Bohr magneton, spin-wave stiffness, and *g*-factor, respectively, and use the following values for Ni: *M*_S_ = 528 kA/m (528 G) [24], *μ*_a_ = 0.6155 *μ*_B_ [25], *ρ*_a_=9.14×10^28^m^−3^, *D*=6.41×10^−40^Jm^2^ (400meVÅ^2^) [26, 27], *g* = 2.21 [28]. With Eq. (3) and the relation [29]

$$A=\frac{D\;\rho_{a}\;\mu_{a}}{2\;g\;\mu_{B}}$$

these parameters suggest *l_M_* = 6.9 nm for the magnetostatic exchange length in Ni. This value is comparable to experimental grain sizes. In Fig. 1, the value of the exchange length of the internal field, *l_H_*, in Ni, is plotted as a function of *H*_i_ for a typically accessible field interval in a SANS experiment. It is seen that for fields between 1 kA/m and 10^4^ kA/m the exchange length varies between about 500 nm and 2 nm. Thus, the interval of exchange lengths is comparable to the interval of length scales that is accessible to measurement by SANS. At low fields *l_H_* is larger than typical grain sizes in nanocrystalline materials, which are of the order of 10 nm, but at the higher end of the field interval *l_H_* is smaller than the grain size. The effective field for Ni is plotted in Fig. 2 as a function of the magnitude of the wavevector ***q***, and of the internal field *H*_i_. It is seen that *H*_eff_ is always large at high *q*, irrespective of the magnetic field. By Eq. (8), this implies that the high-*q* Fourier components of the magnetization are small, in other words sharp variations in ***M*** are suppressed by the effective field. Increasing *H*_i_ has a significant effect on *H*_eff_ only at low *q.* Therefore only the low-*q* Fourier components of the magnetization are suppressed when *H*_i_ is increased, and the long-range variations in ***M*** are reduced. An explicit solution for the spatial variation of ***M*** for a special case is presented in Sec. 6.1 below.

For use in discussion of neutron scattering in Sec. 3, it is of interest to compute |***m***|^2^. With Eq. (8), this is found to be

$$\begin{array}{c} {\left|m(q)\right|}^{2}=\frac{{\left|h(q)\right|}^{2}}{{\left(H_{\mathrm{eff}}+M_{S}\;\sin^{2}\theta \right)}^{2}}\\ +\frac{{\left|h(q)\times q_{⊥}\right|}^{2}}{q^{2}}\\ \times \frac{M_{S}\;(2H_{\mathrm{eff}}+M_{S}\;\sin^{2}\theta )}{H_{\mathrm{eff}}^{2}\;{\left(H_{\mathrm{eff}}+M_{S}\;\sin^{2}\theta \right)}^{2}} \end{array}$$ (12)

for arbitrary orientation of ***q***. We find it convenient to express the vector ***h*** in terms of the scalar anisotropy field amplitude *h*(***q***) = |***h***(***q***)| and of a suitable variable for the orientation of ***h***. In the following, ***H***_i_ and ***M*** will be considered along ***e****_z_*, so that ***H***_p_ and, hence, ***h***, are confined in the plane containing ***e****_x_* and ***e****_y_*. The orientation of ***h*** can then be specified by the angle, *ψ*, included by ***h*** and ***e****_x_*. In terms of these quantities, Eq. (12) is

$${\left|m(q)\right|}^{2}=h{\left(q\right)}^{2}\;F(\psi ,\;q,\;H_{i}).$$ (13)

In other words, |***m***(***q***)|^2^ is proportional to the magnitude square of the anisotropy field and to a scalar function *F* that depends on the vector ***q*** and (through the effective field) on the vector ***H***_i_, as well as on their orientations relative to ***h***(***q***).

## 3. Magnetic Small Angle Neutron Scattering

In this section, we derive a general equation for elastic SANS by micromagnetics structures that describes the differential scattering cross-section as the product of two functions: one dependent on the anisotropy field microstructure alone, and thus independent of the applied magnetic field, and a second that accounts for the field dependent response of the magnetization to the anisotropy field.

The macroscopic differential scattering cross-section (per volume) for elastic magnetic scattering at scattering vector ***k***, due to an arrangement of atoms with positions ***x****_j_*, occupying a total volume of *V*, is the square of the magnitude of the sum of the atomic scattering amplitudes, including phase-shifts that depend on the orientation of the moments and on the atomic position [6, 31–33]:

$$d\Sigma_{\mathrm{mag}}(k)/d\Omega ={\left|\Sigma_{j}b_{\mathrm{mag},j}\;Q_{j}\;\exp(ikx_{j})\right|}^{2}/V.$$ (14)

Here, *b*_mag_ denotes the magnetic scattering length of a single atom with magnetic moment ***μ***_a_, and the vector ***Q*** is related to a unit vector *ε* in the direction of ***k*** and to the atomic magnetic moment by the vector function

$$Q=\varepsilon \;(\varepsilon \cdot \frac{\mu_{a}}{\mu_{a}})-\frac{\mu_{a}}{\mu_{a}},$$ (15)

which is alternatively and equivalently expressed by the Halpern-Johnson tensor [31].

It is well known that the discreteness of the atomic structure of matter is of no importance for small-angle scattering. Therefore, the sum in Eq. (14) can be replaced by an integral involving the magnetization and the phase factor; this leads to an expression for the differential scattering cross-section in terms of the Fourier transform of the magnetization [33]. It is also well known that adding an arbitrary constant to all the vectors ***Q*** leaves the scattering cross-section invariant, except for additional forward scattering which is not relevant to experiment. Since ***Q*** is a linear vector function of the magnetization, replacing ***μ***_a_/***μ***_a_ in Eq. (15), or the equivalent continuous function ***M***(***x***)/*M*_S_, by the reduced perpendicular component of the magnetization ***M***_P_(***x***)/*M*_S_ amounts to such a change of ***Q*** by an additive constant vector. Since ***M***_P_(***x***)/*M*_S_ is the Fourier transform of ***m***(***q***) [compare to Eq. (6)], evaluation of the integral equivalent to Eq. (14) leads to

$$d\Sigma_{\mathrm{mag}}(k)/d\Omega =8\pi^{3}\;V^{-1}\;b_{\mathrm{mag}}^{2}\rho_{a}^{2}{\left|p(k)\right|}^{2},$$ (16)

where *ρ*_a_ is assumed to be uniform, and ***p***(***k***) is defined by

p(k)=ε[ε⋅m(k)]−m(k). (17)

Eq. (17) implies |***p***(***k***)|^2^ = |***m***(***k***)|^2^ sin^2^ *α*, with *α* the angle included by ***m*** and ***k***. Equation (16) is therefore formally identical to well known results for magnetic neutron scattering (e.g., Ref. 33), except that it expresses the scattering cross-section in terms of the Fourier transform of ***M***_P_ instead of ***M***.

When the magnetization obeys the linearized micromagnetics solution of the previous section, then Eq. (13) implies that the expression for the differential scattering cross-section, Eq. (16), can be re-written as the product of an *anisotropy field scattering function S_H_*(***k***), that depends only on the anisotropy field, hence on the nuclear microstructure, but not on the applied field, and of a micromagnetic response function for SANS, *R*(*ψ*, ***k***, ***H***_i_), that depends on the applied field and on the scattering vector, as well as on the relative orientations of these quantities, but not on the geometry of the microstructure:

$$d\Sigma_{\mathrm{mag}}(k)/d\Omega =S_{H}(k)\;R(\psi ,\;k,\;H_{i}).$$ (18)

It is convenient to define *S_H_* and *R* so that *R* is a dimensionless function, and so that *S_H_* has the same units as d*∑*_mag_/d*Ω*:

$$S_{H}(k)=8\pi^{3}\;V^{-1}\;b_{\mathrm{mag}}^{2}\rho_{a}^{2}\;h\;{\left(k\right)}^{2}/M_{S}^{2},$$ (19)

$$R(\psi ,k,H_{i})=M_{S}^{2}\;F(\psi ,k,H_{i})\;\sin^{2}a.$$ (20)

Besides making *R* dimensionless, the inclusion of terms $M_{S}^{2}$ in the definitions of both *S_H_* and *R* has the additional benefit of making *S_H_*, which is related to the anisotropy field, not to the magnetization, actually independent of the atomic magnetic moment. This follows since, by definition, *M*_S_ = ***μ***_a_***ρ***_a_, and since *b*_mag_ = 0.27 × 10^−14^ m *f* ***μ***_a_/***μ***_B_, where *f* denotes a form factor with *f* = 1 in the small-angle scattering region [6]. The anisotropy field scattering function is therefore, equivalently to Eq. (19), expressed in terms of the constant *b_H_* which does not depend on the material:

$$S_{H}(k)=8\pi^{3}\;V^{-1}\;b_{H}^{2}\;h\;{\left(k\right)}^{2},$$ (21)

$$b_{H}=0.27\times 10^{-14}m/\mu_{B}.$$ (22)

In SI units, *b_H_* = 2.9 × 10^8^ A^−1^ m^−1^ (in cgs units *b_H_* = 2.3 × 10^10^ Oe^−1^ m^−2^).

Equations (18)–(22) are central results of this work. Within the limits of applicability of the linearized micromagnetics equation, hence of the results of Sec. 2, they imply that the field-dependent magnetic scattering cross-section for neutrons depends on the microstructure through a single function, *S_H_*(***k***). When the saturation magnetization and exchange stiffness constant are known, then the response function can be computed, and the equations then allow the anisotropy field scattering function to be determined from experimental scattering data, thus enabling measurement of the anisotropy field microstructure.

The explicit general expression for *R*(*ψ*, ***k***, ***H***_i_) in terms of the magnitudes and angles of the quantities involved is lengthy and not illuminating, and it is therefore preferred to display results for some special geometries and averages that are of experimental interest.

## 4. Explicit Results for Scattering by Isotropic Microstructures in Special Scattering Geometries

Two averages are often relevant to experiment: the first extends over the scattering intensities of several defects that are statistically uncorrelated, and the second is an azimuthal average of the intensity on the detector. In considering the first average, we assume that the Fourier coefficients of the anisotropy field can be expressed as

$$h(q)=\Sigma_{j}\;h_{j}(q),$$ (23)

with the ***h****_j_*(***q***) originating from individual defects (e.g., grains). Attention is restricted to microstructures where the directions of the anisotropy fields of the individual defects are uncorrelated, so that terms ***h****_i_*(***q***) · ***h****_j_*(***q***) with *i*≠*j* take both signs with equal probability. Consequently, the expectation value for the sum over these terms vanishes, and

$${\left|h(q)\right|}^{2}=\Sigma_{j}{\left|h(q)\right|}^{2}.$$ (24)

Because Eqs. (8) and (17) express ***m*** and ***p*** as linear vector functions of ***h*** and ***m***, respectively, it follows also that

$${\left|m(q)\right|}^{2}=\Sigma_{j}{\left|m_{j}(q)\right|}^{2},\;{\left|p(k)\right|}^{2}=\Sigma_{j}{\left|p_{j}(k)\right|}^{2}.$$ (25)

By comparing Eq. (25.2) with Eq. (16) for the differential scattering cross-section, it is readily verified that the contributions of the individual defects to the overall differential scattering cross-section are also additive:

$$d\Sigma_{\mathrm{mag}}(k)/d\Omega =\Sigma_{j}\;S_{H,j}\;(k\;)R_{j}(\psi ,k,H_{i}),$$ (26)

with $S_{H,j}(k)=8\pi^{3}\;V^{-1}b_{H}^{2}h_{j}{\left(k\right)}^{2}$. For nanocrystalline solids, this additivity of the magnetic scattering associated with the individual grains contrasts with nuclear scattering, where interparticle interference is strong, to the point that a decomposition of the overall nuclear scattering cross-section into a sum over cross-sections of individual grains, similar to Eq. (26), would be meaningless [34].

For microstructures with a high number of defects in the total scattering volume the sum in Eq. (26) can be replaced by an integral over the orientation of the defects. This is conveniently done in terms of a distribution function *s*(***k***, *ψ*), defined so that

$$\Sigma_{l}S_{H,l}(k)=s(k,\psi )\delta \psi ,$$ (27)

the sum being over all defects with ***h****_l_* (***q***) oriented in the interval [*ψ*−*δψ*/2, *ψ*+*δψ*/2].

The response function *R* has comparatively simple representations in terms of the magnitude and orientation of ***k*** when attention is restricted to two particular scattering geometries: the first has the incident neutron wavevector along ***e****_x_*, normal to the applied field, and hence has the scattering vector in the plane containing ***e****_y_* and the direction of the field, ***e****_z_*. In this geometry, the azimuthal angle *φ* under which the scattering is recorded on the two-dimensional detector coincides with the angle included by ***k*** and ***H***_a_, denoted above by *θ*. The second geometry has the incident neutron wavevector along ***e****_z_*, parallel to the applied field. For that geometry, we take *φ* measured relative to ***e****_x_*. With the response functions for the two scattering geometries denoted, respectively, by *R*_⊥_ (*ψ*, *φ*, *k*, *H*_i_) and *R*_‖_ (*ψ*, *φ*, *k*, *H*_i_), one obtains

$$\begin{array}{l} R_{⊥}(\psi ,\varphi ,k,H_{i})=\frac{M_{S}^{2}}{H_{\mathrm{eff}}^{2}}\cos^{2}\psi \\ +\frac{M_{S}^{2}}{{\left(H_{\mathrm{eff}}+M_{S}\;\sin^{2}\varphi \right)}^{2}}\;\sin^{2}\psi \cos^{2}\varphi , \end{array}$$ (28)

$$R_{∥}(\psi ,\varphi ,k,H_{i})=\frac{M_{S}^{2}}{H_{\mathrm{eff}}^{2}}\;\sin^{2}(\varphi -\psi )$$ (29)

In terms of the quantities introduced by Eqs. (27)–(29), the overall differential scattering cross-section obeys

$$d\Sigma_{\mathrm{mag}}(k)/d\Omega =\int_{0}^{2\pi }s(k,\psi )R(\psi ,\varphi ,k,H_{i})d\psi .$$ (30)

The integral has comparatively simple closed-form solutions when *s* is isotropic with respect to *ψ*, that is, when *s*(***k***, *ψ*) = *S_H_*(***k***)/2π:

$$d\Sigma_{\mathrm{mag}}(k)/d\Omega =S_{H}\;(k)\;R_{\mathrm{iso}}(\varphi ,k,H_{i}),$$ (31)

$$\begin{array}{l} R_{\mathrm{iso},⊥}(\varphi ,k,H_{i})=\frac{M_{S}^{2}}{2H_{\mathrm{eff}}^{2}}\\ \left[1+\frac{\cos^{2}\varphi }{{\left(1+\frac{M_{S}}{H_{\mathrm{eff}}}\;\sin^{2}\varphi \right)}^{2}}\right], \end{array}$$ (32)

^1^

$$R_{\mathrm{iso},∥}(\varphi ,k,H_{i})=\frac{M_{S}^{2}}{2H_{\mathrm{eff}}^{2}}.$$ (33)

**Fig. 3:**
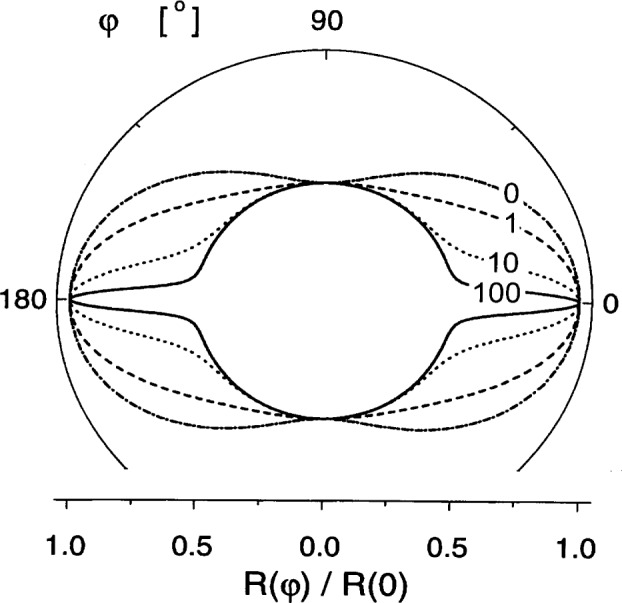
Polar plot of the micromagnetics response function for SANS of isotropic microstructures with neutron beam normal to the magnetic field, *R*_iso,⊥_(*φ*, *k, H*_i_), versus azimuthal angle *φ*. The numbers in the figure indicate the value of the parameter *p* = *M*_S_/*H*_eff_.

Consistent with the symmetry of the arrangement, the scattering pattern has azimuthal isotropy when the field is parallel to the neutron beam [Eq. (33)], but when the field is normal to the beam [Eq. (32)] then the scattering can be highly anisotropic, with the detailed nature of the anisotropy dependent on the value of the parameter *p* = *M*_S_ / *H*_eff_ and, hence, on *k* and *H*_a_. The polar plot of *R*_iso_ for that geometry, Fig. 3, illustrates the anisotropy for different values of *p.* It is immediately obvious that, at all fields, the dependency of d*∑*_mag_/d*Ω* on azimuthal angle is quite different from the well known sin^2^*φ* variation that is observed for a magnetically aligned array of isolated particles in a nonmagnetic matrix, or for an array of pores in a saturated ferromagnetic matrix. The azimuthal anisotropy of d*∑*_mag_/d*Ω* in Fig. 3 at large effective field (small *p*) is readily rationalized in terms of a distribution of ***m***(*k*) that is isotropic in the plane normal to ***H***_a_. At smaller field (large *p*), the scattering cross-section is seen to develop a “spike” in the direction parallel to the field that is explained as follows: for the limit where *M*_S_»***H***_eff_, Eq. (8) shows that ***m***(***k***) can have a significant magnitude only in the direction where ***m***⊥***k***. The suppression of components of ***m*** parallel to ***k*** is a consequence of the demagnetizing field from divergence of the magnetization [compare to Eq. (7)]. Besides ***m***⊥***k***, ***m*** needs also to satisfy ***m***⊥***H***_a_, and for a *general* orientation of ***k*** in the plane normal to ***k***_0_, that contains ***H***_a_, the two conditions for the orientation of ***m*** can only be satisfied simultaneously when ***m*** takes one of two discrete orientations, namely, ***m*** parallel or antiparallel to ***k***_0_. Scattering from these Fourier components with ***m***∥***k***_0_ leads to the circular part of d*∑*_mag_/d*Ω* in the polar plot for large *p.* But for the *special* orientation where ***k***∥***H***_a_, all orientations of ***m*** in the plane normal to ***H***_a_ satisfy ***m***⊥***k***, and can therefore have significant values. For this orientation of the scattering vector, d*∑*_mag_/d*Ω* is therefore not only from two orientations of ***m***, but from the full angular spectrum; hence the spike of higher intensity.

In addition to the average over the orientations of the anisotropy field, one is often interested in the azimuthal average of the scattering cross-section $d{\bar{\Sigma }}_{\mathrm{mag}}(k)/d\Omega ={\left(2\pi \right)}^{-1}\int_{0}^{2\pi }d\Sigma_{\mathrm{mag}}(k)/d\Omega \;d\varphi$. The asymmetry of the microstructure is further restricted by considering only cases where *S_H_* depends only on the magnitude of ***k***, not on *φ*. This applies, e.g., to untextured polycrystals with equiaxed grains or with elongated grains with isotropic orientation distribution of the long axes. Integration of Eqs. (31)–(33) for the isotropic case then yield

$$d{\bar{\Sigma }}_{\mathrm{mag}}(k)/d\Omega =S_{H}\;(k)\;{\bar{R}}_{\mathrm{iso}}(k,H_{i})$$ (34)

$$h\;{\bar{R}}_{\mathrm{iso},⊥}(k,H_{i})=\frac{M_{S}^{2}}{4H_{\mathrm{eff}}^{2}}\left[2+\frac{1}{\sqrt{1+\frac{M_{S}}{H_{\mathrm{eff}}}}}\right],$$ (35)

$${\bar{R}}_{\mathrm{iso},∥}(k,H_{i})=\frac{M_{S}^{2}}{2H_{\mathrm{eff}}^{2}}.$$ (36)

**Fig. 4:**
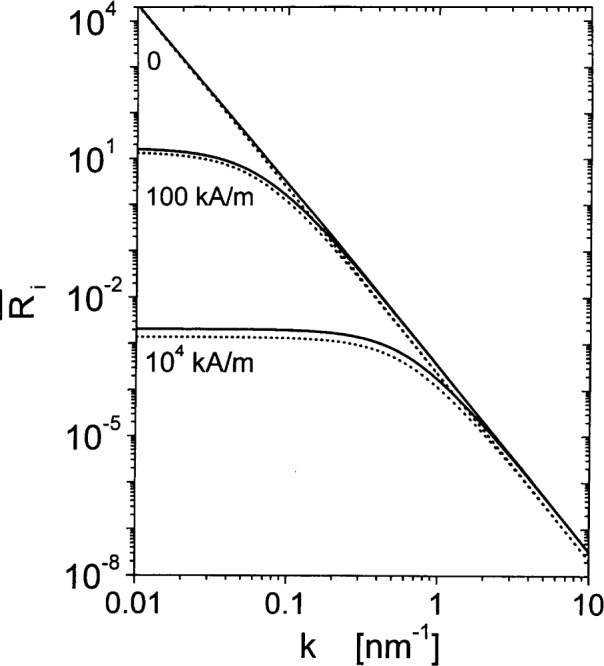
Log-log plot of the micromagnetics response functions for SANS of isotropic microstructures, for neutron beam normal to the field, ${\bar{R}}_{\mathrm{iso},⊥}(k,H_{i})$, (solid lines); and for neutron beam parallel to the field, ${\bar{R}}_{\mathrm{iso},∥}(k,H_{i})$, (dotted lines), versus scattering vector ***k***. Values of magnetic field *H*_i_ are indicated in the figure.

Figure 4 displays the response functions ${\bar{R}}_{\mathrm{iso},⊥}(k,H_{i})$ and ${\bar{R}}_{\mathrm{iso},∥}(k,H_{i})$ at different applied fields, for the example of Ni.

The results for the response function derived in this section can be combined with measured values for the magnetic field *H*_i_, with estimates for the demagnetizing field based on known sample shape, and with known values of the materials parameters, the exchange stiffness constant *A* and saturation magnetization *M*_S_ to explicitly compute the response function. The anisotropy field scattering function can then be computed from experimental scattering data.

## 5. A Sum Rule for the Anisotropy Field Scattering Function

In studies of nuclear scattering, one can often obtain useful information from an invariant of nuclear scattering: the second moment of the radially averaged scattering intensity depends only on the root-mean-square of the variation in scattering length density, but not on the detailed geometry of the microstructure. Here, a similar expression is derived for the anisotropy field scattering function *S_H_*(***q***). The procedure is quite analogous to that applicable to nuclear scattering [35], and is outlined here merely to confirm its applicability to a *vector* function, the anisotropy field, as opposed to the *scalar* nuclear density.

The square of the magnitude of the anisotropy field Fourier coefficient |***h***(***q***)|^2^ is related to ***H***_p_(***x***) in real space by

$$\begin{array}{c} |h(q){|}^{2}=(2\pi {)}^{-3}\int \int \int_{-\infty }^{\infty }H_{p}(x)\;\exp\;(-i\mathrm{qx}\;)d^{3}x\\ \int \int \int_{-\infty }^{\infty }H_{p}(x)\;\exp\;(+i\mathrm{qx})\;d^{3}x. \end{array}$$ (37)

The right-hand side of this equation can be expressed in terms of the Fourier transform of the Patterson or auto-correlation function [35,36] *C* (***r***) of the anisotropy field:

$${\left|h(q)\right|}^{2}={\left(2\pi \right)}^{-3}\int \int \int_{-\infty }^{\infty }C(r)\;\exp\;(i\mathrm{qr})\;d^{3}r,$$ (38)

$$C(r)=\int \int \int_{-\infty }^{\infty }\;H_{p}(x+r)\;H_{p}\;(x)\;d^{3}x.$$ (39)

The back transform of Eq. (38) is

$$C(r)=\int \int \int_{-\infty }^{\infty }{\left|h(q)\right|}^{2}\;\exp\;(-i\mathrm{qr})\;d^{3}q.$$ (40)

When |***h***(***q***)|^2^ is isotropic in the sense that it depends only on the magnitude of ***q***, then evaluation of Eqs. (39) and (40) at ***r*** = 0 leads to a relation for the mean square anisotropy field 〈|***H***_P_(***x***)|^2^〉*_V_*, defined by

$${\langle {\left|H_{P}\right|}^{2}\rangle }_{V}=V^{-1}\int \int \int_{-\infty }^{\infty }{\left|H_{p}(x)\right|}^{2}d^{3}x,$$ (41)

in terms of the measurable function *S_H_*(*q*):

$$\begin{array}{l} {\langle {\left|H_{P}\right|}^{2}\rangle }_{V}=V^{-1}\int_{0}^{\infty }h{\left(q\right)}^{2}4\pi \;q^{2}\;dq\\ =(2\pi^{2}\;b_{H}^{2}{)}^{-1}\int_{0}^{\infty }S_{H}\;(k)\;k^{2}dk. \end{array}$$ (42)

The integrals in that equation are invariants of magnetic scattering that depend only on the mean square anisotropy field but not on the applied field or on the details of the microstructure.

## 6. Results for a Nanocrystalline Material

### 6.1 Micromagnetics Model

In this section we derive a solution for the magnetization in real space in a nanocrystalline material with spherical grains. Based on this solution, criteria are derived for the minimum applied field necessary to warrant the validity of the small misalignment approximation, that is, for *M*_P_/*M*_S_«1.

Consider a single-phase, single component nanocrystalline material where the crystallites have random crystallographic orientation, and where the anisotropy field arises from the magnetocrystalline anisotropy alone. Because each grain “*j*” is a single crystal, the anisotropy field in the grain is a constant vector, ***H***_P,_ *_j_*; between any pair of grains there is a random jump in the direction of the anisotropy field. Since the directions of the ***H***_P,_ *_j_* are uncorrelated, ***h***(***q***) obeys Eq. (24), that is, the mean-square anisotropy field amplitude of the microstructure is a weighted sum of the mean-square anisotropy field amplitudes of the individual grains. The computation of |***h***(***q***)|^2^ for an arbitrary arrangement of grains is therefore straightforward once the solution for the single grain case is known. Therefore, we shall proceed to derive an expression for the anisotropy field amplitude of a single grain, assuming the most simple grain shape, the sphere.

For a sphere with radius R and constant ***H***_P_, the definition of ***h***(***q***) as the Fourier transform of ***H***_P_(***x***) suggests that

$$\begin{array}{r} h_{S}(q,ℛ)=(2\pi {)}^{-3/2}H_{P}\int \int \int \;\exp\;(i\mathrm{qx})d^{3}x\\ =3(2\pi {)}^{-3/2}H_{P}V_{S}\frac{\left[\;\sin(qℛ)-qℛ\cos(qℛ)\right]}{{\left(qℛ\right)}^{3}}, \end{array}$$ (43)

with *V*_S_ the volume of the sphere. Except for the pre-factors, Eq. (43) agrees with a well-known result in the theory of nuclear scattering [36].

For a single ferromagnetic spherical inclusion in a uniform ferromagnetic matrix where the anisotropy field vanishes everywhere outside of the inclusion, the Fourier transform ***m***(***q***) of the magnetization is obtained by inserting the result for ***h***_S_(***q***) into Eq. (8). We could not find exact closed-form solutions for the magnetization in real space, that is, for the inverse Fourier transform of ***m***(***q***). However, an approximate closed-form solution is obtained when the terms in Eq. (8) that are due to the demagnetizing field from divergence of ***M*** are neglected, so that ***m***(***q***) ***= h***(***q***)/*H*_eff_. This approximation is valid when *H*_eff_*»M*_S_. At smaller *H*_eff_ the results constitute upper bounds for the magnitudes of ***m*** and ***M***_P_, because the demagnetizing field always reduces the magnitude of ***m***. With the above assumption, the inverse Fourier transform of ***m***(***q***) yields a magnetization that depends on position only through the scalar distance *r* from the center of the inclusion:

$$\begin{array}{c} M_{P}(r)=g(r,ℛ,l_{H})\;M_{S}\;\frac{H_{p}}{H_{i}},\\ g(r,ℛ,l_{H})=1-\left(\frac{ℛ}{l_{H}}+1\right)\;\exp\left(-\frac{ℛ}{l_{H}}\right)\;\sinh\;\left(\frac{r}{l_{H}}\right)\frac{l_{H}}{r}\\ \text{when}\;r<ℛ, \end{array}$$ (44)

and

$$\begin{array}{c} g(r,ℛ,l_{H})=\left[\frac{ℛ}{l_{H}}\;\cosh\;\left(\frac{ℛ}{l_{H}}\right)-\sinh\;\left(\frac{ℛ}{l_{H}}\right)\right]\;\exp\;\left(-\frac{r}{l_{H}}\right)\frac{l_{H}}{r}\\ \text{when}\;r<ℛ. \end{array}$$ (45)

**Fig. 5:**
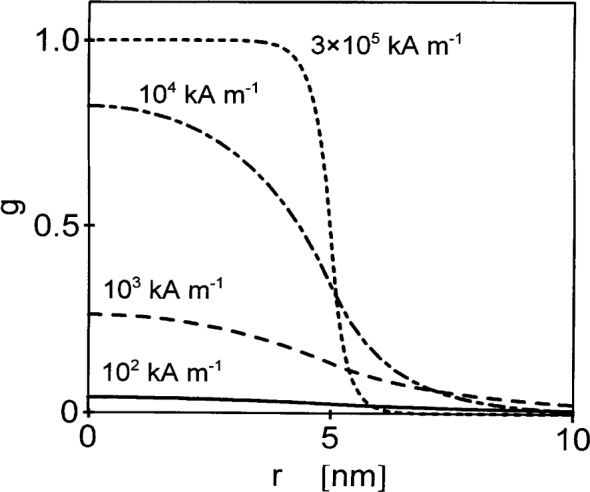
The function *g* for an inclusion with radius ℛ=5 nm in Ni, versus distance *r* from the center of the inclusion. The numbers in the figure indicate the value of the magnetic field *H*_i_.

**Fig. 6:**
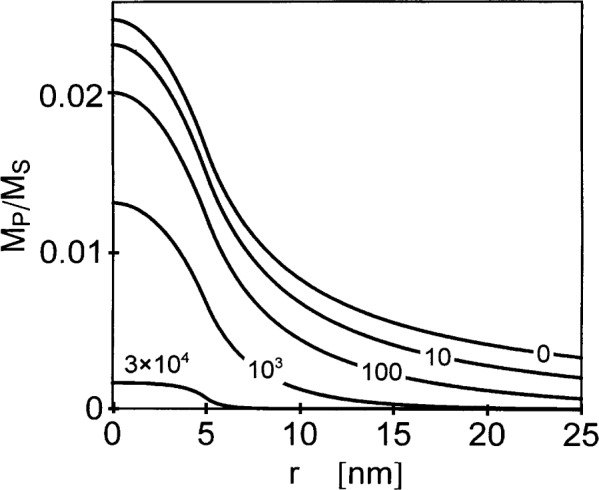
The reduced component *M*_P_/*M*_S_ of the magnetization normal to the applied field for a spherical inclusion of radius ℛ=5 nm in an otherwise anisotropy field-free matrix versus distance *r* from the center of the inclusion. The numbers in the figure indicate the value of the magnetic field *H*_i_ in units of kA/m. Material parameters are for Ni.

The function *g* describes the response of the magnetization to the anisotropy field in the inclusion. Figure 5 displays *g* for different values of the internal field, and for the example of an inclusion in Ni with ℛ = 5 nm, corresponding to a grain size of 10 nm. It is seen that at high magnetic fields *g* varies steeply at the interface between inclusion and matrix; as the field is decreased, the variation at the interface is smeared out. Figure 6 shows the magnitude of the normal component of the magnetization *M*_P_ versus *r.* In Fig. 6, *H*_P_= 10^−2^ T, and the remaining parameters are the same as in Fig. 5. Consistent with Fig. 5, it is seen that at high fields, when *l_H_* «ℛ, the variation of the magnetization is confined to a narrow region near the interface between inclusion and matrix, and that at lower fields the magnetization varies in a transition region which extends on a larger scale into the inclusion and into the matrix.

**Fig. 7:**
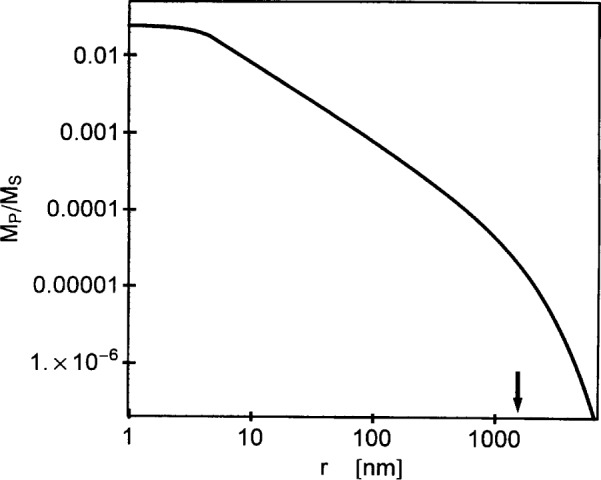
Log-log plot of reduced normal magnetization *M*_P_/*M*_S_ versus distance *r* from the center of a spherical inclusion with radius ℛ=5 nm in Ni, at an internal field of 10^−2^ kA/m. The arrow indicates the exchange length *l_H_*.

Consider the case of small applied fields, where *l_H_* > ℛ. In this case, inspection of Eq. (44) shows that there is a “slow” decrease of *M*_P_ with distance *r* from the center of the inclusion in the region outside the inclusion where ℛ < *r*< *l_H_*, with approximately *M*_P_ ∝ 1/*r*. A faster, approximately exponential decrease is suggested by the same equation for larger distances, *r* > *l_H_*. This is illustrated in the log-log plot of *M*_P_ versus *r* in Fig. 7, where the 1/*r* variation of *M*_P_ leads to a straight line for ℛ < *r* < *l_H_*. The transition between slow and fast decrease of *M*_P_ at *r* ≈ *l_H_* suggests that variations of ***M*** are correlated on a length-scale of the order of *l_H_.* Since *l_H_* varies as the inverse root of the applied field, this correlation length diverges when *H_i_* is reduced to zero (compare to Fig. 1). This is also seen from the limiting-form of Eq. (44) for *H_i_* = 0, which has a 1/*r* variation of the normal component of ***M*** everywhere outside the inclusion:

$$M_{p}(r)=H_{p}\frac{3{ℛ}^{2}-r^{2}}{6\;l_{M}^{2}}\;\text{when}\;r<ℛ,H_{i}=0,$$ (46)

and

$$M_{p}(r)=H_{p}\frac{{ℛ}^{3}}{3\;l_{M}^{2}r}\;\text{when}\;r>ℛ,\;H_{i}=0.$$

Equation (46) suggests that, in the case of a single inclusion in an otherwise uniform matrix, the condition *H_p_* «2 *M*_S_ $l_{M}^{2}/{ℛ}^{2}$ is sufficient for the misalignment to be small and the linearized theory to be applicable, independent of the applied field. For Ni with a grain size of 10 nm, this requires *H*_P_«2000 kA/m, and compares to considerably smaller expectation values for *H*_P_ due to magnetocrystalline anisotropy of about 3.7 kA/m at 300 K and 74 kA/m at 4 K (compare to Sec. 6.2 below).

Let us now consider the validity of the small misalignment approximation in the case of a nanocrystalline material, that is, a material entirely occupied by grains such as the one discussed above. At internal fields sufficiently large that *l_H_* «*ℛ* (or, equivalently, *H*_i_*»M*_S_ $l_{M}^{2}/{ℛ}^{2}$), the perpendicular magnetization decays exponentially outside each grain, so that there is little overlap of magnetization profiles from neighboring grains. However, at small applied fields, *l_H_*, and hence the range of the perturbations, are larger than the grain size (*l_H_* «*ℛ* or, equivalently, *H*_i_*»M*_S_ $l_{M}^{2}/{ℛ}^{2}$). In this case, the net value of the perpendicular magnetization at a given point is a superposition of perturbations with random sign originating from a large number of neighboring grains. Therefore, even when ***M***_P_ due to the anisotropy field of each individual grain is small, the expectation value for the net magnitude of ***M***_P_ may be large. As a measure of the mean net misalignment in the nanocrystalline material we consider the volumetric mean square of ***M***_P_, defined by ${\langle {\left|M_{P}\right|}^{2}\rangle }_{V}=V^{-1}{\int |M_{P}|}^{2}dV$, with the integral extending over the entire volume of the material. Considerations analogous to those leading to Eq. (25) suggest that the contributions of individual grains to the integral are additive, so that ${\langle {\left|M_{P}\right|}^{2}\rangle }_{V}=V^{-1}\sum_{j}{\left|M_{p,j}(r)\right|}^{2}4\pi r^{2}\mathrm{dr}$, the summation being over the individual magnetization profiles of all grains. With Eq. (44) for ***M***_P_, this leads to

$${\langle {\left|M_{P}\right|}^{2}\rangle }_{V}={\langle {\left|H_{P}\right|}^{2}\rangle }_{V}\frac{l_{H}^{4}}{l_{M}^{4}}\left[1-\frac{9}{4}\frac{l_{H}}{ℛ}+\frac{15}{4}\frac{l_{H}^{3}}{{ℛ}^{3}}-\frac{3}{4}\;\exp\left(-2\frac{ℛ}{l_{H}}\right)\left(2+7\frac{l_{H}}{ℛ}+10\frac{l_{H}^{2}}{{ℛ}^{2}}+5\frac{l_{H}^{3}}{{ℛ}^{3}}\right)\right],$$ (47)

and to the limiting forms

$${\langle {\left|M_{P}\right|}^{2}\rangle }_{V}=\frac{1}{6}{\langle {\left|H_{P}\right|}^{2}\rangle }_{V}\frac{ℛ}{l_{M}^{3}}\sqrt{\frac{M_{S}}{H_{i}}},\text{when}\;l_{H}»ℛ,$$ (48.1)

and

$${\langle {\left|M_{P}\right|}^{2}\rangle }_{V}=M_{S}^{2}\frac{{\langle {\left|H_{P}\right|}^{2}\rangle }_{V}}{H_{i}^{2}},\;\text{when}\;l_{H}«ℛ.$$ (48.2)

Expressing the requirement of small misalignment, somewhat arbitrarily, as 〈|***M***_P_|^2^〉*_V_*/***M***_S_^2^ < 0.01, it is found from these results that small misalignment requires $H_{i}>300\;{\langle {\left|H_{P}\left(x\right)\right|}^{2}\rangle }_{V}^{2}\;M_{S}^{-3}{\left(ℛ/l_{M}\right)}^{6}$ when *l_H_* » *ℛ*, and $H_{i}>10\;{\langle {\left|H_{P}\left(x\right)\right|}^{2}\rangle }_{V}^{1/2}$ when *l_H_* «*ℛ*. For the example of Ni with a grain size of 10 nm and *H*_P_ = 50 kA/m, the two conditions are *H*_i_ > 1.9 kA/m and *H*_i_ > 500 kA/m, respectively. The second condition is automatically satisfied since, by Eq. (10)*l_H_* < *ℛ* implies *H*_i_ > 1000 kA/m. In conjunction with the first condition this implies that, for the example, the small misalignment approximation remains valid down to quite small applied fields.

Since the magnitude of the mean (macroscopic) magnetization in the model is approximately

$$\left|{\langle M\rangle }_{V}\right|=M_{S}-\frac{{\langle {\left|M_{P}\right|}^{2}\rangle }_{V}}{2M_{S}},$$

Eq. (47) has an immediate relation to the approach to saturation in a magnetization isotherm. Results for the magnetization of amorphous ferromagnets with random anisotropy are formally similar to the expression for the mean magnetization implied by Eq. (47), and compare favorably to experimental magnetization isotherms of nanocrystalline ferromagnets [22, 37, 38]. A discussion with relation to experimental magnetization data for nanocrystalline Ni and Co will be given in a subsequent publication [20].

### 6.2 SANS

As above, we consider a nanocrystalline material with magnetocrystalline anisotropy only, and with random crystallographic orientations of the grains. The scattering cross-section will then depend on the mean-square anisotropy field and on the grain size or the distribution of sizes. Because of the random orientation, the expectation value for |***H***_P_|^2^ in a grain is independent of the grain size, and is identical to the value of the volumetric mean-square anisotropy field. The expectation value is obtained by computing ***H***_P_ in a single crystal as a function of the orientation of the magnetization relative to the crystal lattice, and averaging |***H***_P_|^2^ over all orientations:

$${\langle {\left|H_{P}\right|}^{2}\rangle }_{\Omega }={\left(4\pi \right)}^{-1}\int {\left|H_{P}\right|}^{2}d\Omega ,$$ (49)

where *Ω* denotes the solid angle. 〈|***H***_P_|^2^〉*Ω* is a materials constant, and is independent of grain size and grain shape. Using the values for the magnetocrystalline anisotropy constants in Refs. [39, 40], one finds that for Ni ${\langle {\left|H_{P}\right|}^{2}\rangle }_{\Omega }^{1/2}=74\mathrm{kA}/m$ at 4 K and ${\langle {\left|H_{P}\right|}^{2}\rangle }_{\Omega }^{1/2}=3.7\mathrm{kA}/m$ at 300 K.

The distribution of grain sizes is described by the function *n*(*ℛ*), defined so that the number of grains with radius in the interval [*ℛ*, *ℛ*+d*ℛ*] is *n*(*ℛ*)d*ℛ*. In analogy to nuclear scattering by noninterfering particles, the anisotropy field scattering function is an integral over the scattering cross-sections of the individual grains, weighted by the grain-size distribution function. For spherical grains with random crystallographic orientations, Eqs. (20) and (43) suggest that the anisotropy field scattering function is then

$$S_{H}(k)=12\pi b_{H}^{2}{\langle {\left|H_{P}\right|}^{2}\rangle }_{\Omega }k^{-6}\times \frac{\int_{0}^{\infty }n(ℛ){\left[\;\sin(kℛ)-kℛ\cos(kℛ)\right]}^{2}dℛ}{\int_{0}^{\infty }n(ℛ){ℛ}^{3}dℛ}.$$ (50)

Except for the prefactors, Eq. (50) is identical to the nuclear interference function of an array of noninterfering particles, and general asymptotic results at small and large *k* are therefore immediately transferable. In particular, the Guinier approximation [35, 36] links *S_H_*(*k*) at small *k* to a mean grain radius ${\bar{ℛ}}_{H}$ according to $S_{H}\left(k\right)\propto \;\exp(–{\bar{ℛ}}_{H}^{2}k^{2}/3)$; and the asymptotic variation of *S_H_*(*k*) at large *k* satisfies the Porod approximation [41] with

$$S_{H}(k)=2\pi b_{H}^{2}{\langle {\left|H_{P}\right|}^{2}\rangle }_{\Omega }k^{-4}A/V,$$ (51)

where A denotes the total grain boundary area.

**Fig. 8:**
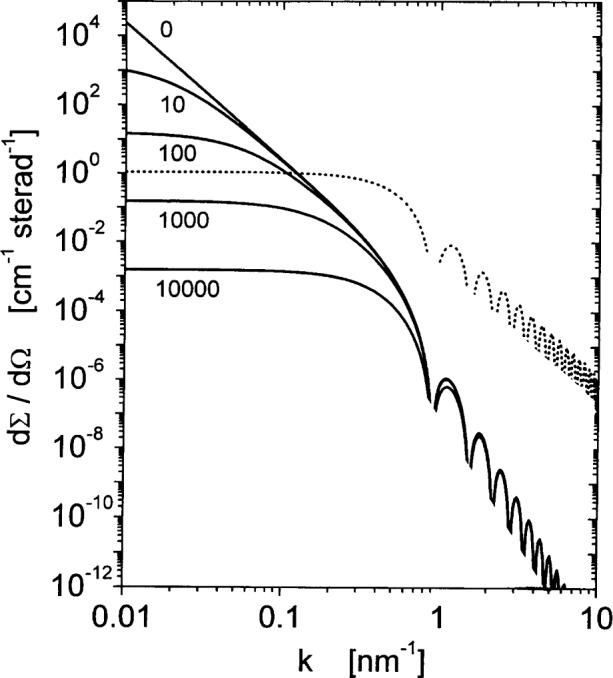
Full lines: magnetic differential scattering cross-section d*∑*_mag_/d*Ω* for nanocrystalline Ni with spherical grains of radius ℛ=5 nm and with ${\langle {\left|H_{P}\right|}^{2}\rangle }_{\Omega }^{1/2}=50\;\mathrm{kA}/m$, plotted versus scattering vector ***k*** for parallel geometry. The numbers indicate values of the magnetic field *H*_i_ in kA/m. Dotted line: anisotropy field scattering function *S_H_*.

Note that information on the nuclear microstructure is here obtained by analysis of *S_H_*(*k*); since the differential scattering cross-section depends on the product of *S_H_*(*k*) and the micromagnetics response function *R*, it is not permissible to derive information on the nuclear microstructure by analyzing d*Σ*_mag_/d*Ω* immediately in terms of the Guinier or Porod approximations. In fact, the asymptotic variation of d*Σ*_mag_/d*Ω* at high *k* is readily seen to by quite different from the *k*^−4^ law: at high *k*, the effective field *H*_eff_ increases as *k*^2^, and hence the response functions vary asymptotically as *k*^−4^. In conjunction with the *k*^−4^ variation of *S_H_*(*k*), this gives rise to d*Σ*_mag_/d*Ω* ∝ *k*^−8^, which is a much steeper dependency than the d*Σ*/d*Ω* ∝ *k*^−4^ intensity variation that is expected for nuclear scattering from microstructures with sharp interfaces. The high power-law exponent at large *k* can be seen in Fig. 8, which shows plots of d*Σ*_mag_/d*Ω* at different magnetic fields for Ni with a monodisperse grain size of 10 nm and with 〈|***H***_P_|^2^〉*_Ω_*^1/2^ = 50 kA/m.

The Guinier radius obtained by analysis of d*Σ*_mag_/d*Ω* depends both on the anisotropy field microstructure and on the applied field. A series expansion of Eq. (36) about *k* = 0 yields ${\bar{ℛ}}_{i}\propto 1-l_{H}^{2}k^{2}$ at small *k*, which implies the asymptotic form $d\Sigma_{\mathrm{mag}}/d\Omega \propto \;\exp[-\left({\bar{ℛ}}_{H}^{2}/3+l_{H}^{2}\right)k^{2}]$. Unless *H*_i_*«M*_S_, the same result is obtained with Eq. (35). Therefore, independent of the scattering geometry, a Guinier fit to d*Σ*_mag_/d*Ω* yields a field-dependent effective Guinier radius that obeys

$${\bar{ℛ}}_{\mathrm{eff}}^{2}=\frac{{\bar{ℛ}}_{H}^{2}}{3}+\frac{2A}{\mu_{0}\;M_{S}\;H_{i}}.$$ (52)

In conjunction with this result, experimental investigation of the field-dependence of ${\bar{ℛ}}_{\mathrm{eff}}$ may provide a means for measuring the exchange constant *A.*

## 7. Summary and Discussion

In summary, we have presented an analysis of small-angle neutron scattering by nanocrystalline ferromagnets that is based on an analysis of the magnetic microstructure in terms of the theory of micromagnetics. The analysis requires small misalignment of the magnetic moments and uniform magnitude of magnetization and exchange interaction; it applies irrespective of the nature of the magnetic anisotropy. Our results for the variation of the differential scattering cross-section with the applied field suggest that SANS experiments carried out at different fields allow the measurement of the anisotropy field scattering function. This function contains information on the magnitude of the anisotropy field and on the length scales over which it is correlated. Because the coefficients of magnetocrystalline and magnetoelastic anisotropy vary independently with temperature, comparison of anisotropy field scattering functions measured at different temperatures can also lead to insight into the nature of the anisotropy.

In experimental studies, the nuclear density and/or composition will generally be nonuniform, and consequently there can be a nonuniformity in the magnetization, even at the highest fields when all spins are aligned. The nuclear scattering cross-section is independent of the applied field and the same holds true, in the limit of small misalignment (small ***M***_P_), for the magnetic scattering due to nonuniform saturation magnetization. This combined nuclear and magnetic *residual scattering cross-section* d*Σ*_residual_/d*Ω* is not accounted for in our micromagnetics approach. When the arrangement of the elements of the nuclear microstructure that give rise to residual scattering is uncorrelated with the arrangement of elements that are responsible for the anisotropy field microstructure, then the two scattering cross-sections are additive, so that the *total* differential scattering cross-section d*Σ*_total_/d*Ω* is

$$\frac{d\sum_{\text{total}}(k,H_{i})}{d\Omega }=\frac{d\sum_{\text{residual}}(k)}{d\Omega }+\frac{d\sum_{\mathrm{mag}}(k,H_{i})}{d\Omega }.$$

Therefore, when the response function is known, measurement of the total scattering cross-section at two different applied fields is required to compute the two unknown functions d*Σ*_residual_(***k***)/d*Ω* and *S_H_*(***k***). Note that the azimuthal anisotropy of the residual scattering cross-section is in general quite different from that of d*Σ*_mag_/d*Ω*. For instance, an isotropic microstructure subject to an applied field orthogonal to the incident beam has $d\Sigma_{\text{residual}}/d\Omega \propto \left[b_{\mathrm{nuc}}^{2}+b_{\mathrm{mag}}^{2}\;\sin^{2}\left(\varphi \right)\right]$, where *b*_nuc_ denotes the atomic nuclear scattering length, whereas d*Σ*_mag_/d*Ω* obeys the quite different dependence on azimuthal angle expressed by Eq. (32).

In addition to their different dependency on the applied magnetic field and on the azimuthal angle, the two contributions to the total scattering cross-section also depend in a different way on change of the neutron polarization state. In residual scattering there is interference between magnetic and nuclear scattering amplitudes; such interference implies that the magnitude of the scattering cross-section will depend on the neutron polarization state [6, 33]. By contrast, there is no interference between the nuclear scattering and the scattering from micromagnetics structures in our model, and consequently d*Σ*_mag_/d*Ω* is invariant with respect to a change of polarization. Experimental studies with polarized neutrons may therefore provide a verification of the separation of residual from micromagnetics scattering.

Spin waves do not give rise to *elastic* scattering, since the cross-section for elastic scattering depends on the time average of the time-dependent correlation function. But in general SANS instrumentation does not completely discriminate *inelastic* scattering; therefore, experimental SANS data may contain contributions due to inelastic scattering from spin waves [42]. With a magnon dispersion relation *ħω* = *D k*^2^ + *gμ*_B_*μ*_0_*H*_i_, and an incident neutron of wavevector ***k***_0_, mass *m*_n_, and energy *ħω* = *ħ*^2^*k*_0_^2^/(2 *m*_n_), the balances of energy and momentum for the inelastic scattering event can only be satisfied simultaneously when

$$\left|\left(\frac{2m_{n}D}{\hbar^{2}}\pm 1\right)\frac{k}{2k_{0}}+\frac{m_{n}g\mu_{B}\mu_{0}H_{i}}{\hbar^{2}k_{0}k}\right|\leq 1.$$

This relation imposes upper and lower limits for the allowed range of scattering vectors *k* for inelastic SANS from spin waves, and implies that this range narrows as the magnetic field *H_i_* is increased. With 2 *m*_n_ *D*/*ħ*^2^»1 for most elemental ferromagnets (the value for Ni is 193), the term ±1 (where + or − refer to the generation or annihilation of a magnon, respectively) may be neglected, and it follows that spin wave scattering is completely suppressed when the applied magnetic field satisfies

$$H_{i}\tilde{>}\frac{\hbar^{4}k_{0}^{2}}{4m_{n}^{2}g\mu_{B}\mu_{0}D}.$$

For Ni and an incident neutron wavelength of 0.6 nm, this requires *H_i_* ⪞ 73 kA/m (920 Oe). Correction of the scattering data for signal from inelastic scattering may be required at smaller magnetic fields.

Our results imply that information on the anisotropy field microstructure may be obtained by analyzing the experimental anisotropy field scattering function: the value of the volumetric mean square anisotropy field can be measured, as can the total grain boundary area per volume and a mean grain radius, in case the magnetic anisotropy is dominated by magnetocrystalline anisotropy. In principle, the anisotropy field scattering function can also be analyzed in terms of a grain size distribution function, quite analogous to the analysis of nuclear scattering data by non-interfering particles. This is of relevance because a similar analysis is not possible for nuclear scattering by bulk nanocrystalline solids [34]. Thus, magnetic SANS may contribute to the characterization of the nuclear microstructure of nanocrystalline solids.

Because of the restrictive assumption of small misalignment, our discussion cannot provide an adequate analysis of the magnetic domain structure that develops at small applied magnetic fields. When the domain size is much larger than the characteristic size of coherent magnetic fluctuations, then it may be expected that the magnetic microstructure inside each domain may still be adequately described by the linearized theory. Also, the theory of micromagnetics applies to the structure of the domain walls, and the dependency of wall width on the grain size has been discussed in the literature [1]. Domain walls contribute to SANS at small applied field, but the magnitude of the signal depends on the wall area per volume, and therefore on the *a priori* unknown domain size. Hence, it is not immediately obvious how the effect of magnetic domains on SANS can be quantified. Previous experimental studies of SANS from nanocrystalline Fe and Ni have inferred a magnetic domain size by analyzing the data in terms of scattering by noninterfering, uniformly magnetized domains [16, 43]. Our results do not support such analysis, since they suggest that the magnetization inside the domains is highly nonuniform. In other words, there is strong scattering due to a superposition of uncorrelated and overlapping perturbations of the magnetization that decorate the anisotropy field microstructure; this scattering is on top of scattering by the magnetic domain structure. The magnetic scattering cross-sections of the individual perturbations can be additive, but their functional form (which depends on the applied magnetic field) is quite different from scattering by uniformly magnetized domains.

Contrary to our assumption of uniform exchange stiffness constant, the magnetic interactions in the core of defects in real materials may differ from those in the bulk. For instance the exchange coupling across grain boundaries may be weakened relative to the bulk, suggesting a jump of the magnetization vector across grain boundaries. The correspondingly larger Fourier components of the magnetization at high wavevector would lead to measurable deviations of the experimental scattering cross-section at high scattering vector from the predictions in the present work. Therefore, experiment may provide a test of the validity of the assumption of uniform exchange stiffness constant.

In subsequent publications [20], we shall present experimental SANS data for electrodeposited nanocrystalline Ni and Co that show good agreement with the predictions of this paper.

## Footnotes

1 Equation (32) corrects an earlier, erroneous result in Ref. [19].
